# 3D-printed syringe holder with synchronized push-pull action

**DOI:** 10.1016/j.ohx.2026.e00756

**Published:** 2026-03-04

**Authors:** Daniel P.G. Nilsson, Magnus Andersson

**Affiliations:** a Department of Physics, Umeå University, Umeå, 901 87, Sweden; b Umeå Centre for Microbial Research, Umeå University, Umeå, 901 87, Sweden

**Keywords:** Push2Pull, Syringe holder, Syringe pump, Culture medium, Fluid exchange, Design-build-test

## Abstract

Controlled liquid exchange is a fundamental requirement for numerous biological and chemical experimental protocols. However, achieving constant-volume exchange often requires electronic syringe pumps that are cost-prohibitive and time-consuming to set up in a push-pull configuration. To address these limitations, we developed the *Push2Pull syringe holder*, a simple 3D-printed device that mechanically synchronizes two syringes to simultaneously add and extract equal fluid volumes. This device is compatible with standard disposable syringes from 1 to 60 mL in size and operates without electricity or additional hardware, making it ideal for both laboratory and field settings. Validation experiments demonstrate an exchange accuracy within ±2%v/v across the whole travel range, while fluid exchange efficiency was calculated for various use cases using CFD simulations. The *Push2Pull syringe holder* offers an accessible, open-source solution for precise fluid handling, for a material cost of less than $10.

## Graphical abstract

**Figure:**
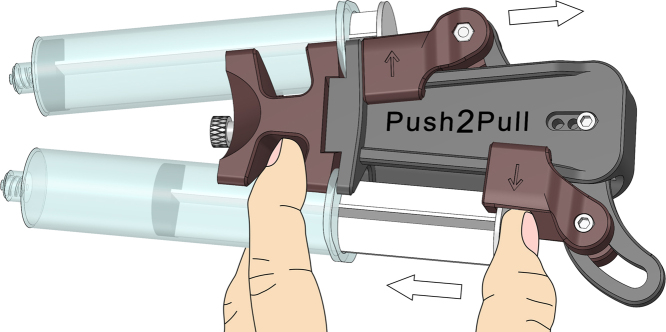

## Specifications table

**Table:** 

**Hardware name**	Push2Pull Syringe Holder

**Subject area**	Biological sciences (e.g. microbiology and biochemistry)

**Hardware type**	Biological sample handling and preparation

**Closest commercial analog**	No commercial analog is available.

**Open source license**	CC BY-NC-SA 4.0

**Cost of hardware**	$10

**Source file repository**	https://doi.org/10.5281/zenodo.17711432

## 1 Hardware in context

Controlled liquid exchange is essential in many biological and chemical workflows, such as cell culture maintenance, staining, and reagent replenishment [1], [2], [3], [4]. These processes often require precise removal and addition of liquids without altering the sample volume, which can be challenging in open containers like culture plates and test tubes, especially when the exchange volume exceeds the container’s spare capacity. Automated cell culture systems have come to solve many of these challenges and replace technicians for some specific tasks where scale and cost allow. However, manual handling still remains the go-to procedure for most research, industry, and educational labs, even though it is prone to volumetric inaccuracies and the risks of overflow.

To mitigate these issues, electronic syringe pumps are often employed in a push–pull configuration, simultaneously adding and extracting equal fluid volumes. While effective, these systems are expensive and time- consuming to set up, typically requiring a computer and power supply to run, as well as hoses and connectors to reach the sample. To address the cost barrier, various 3D-printable syringe pumps have been developed during the past decade, with build costs starting around $100 [5], [6], [7], [8], [9], [10]. However, these designs retain the complexity of commercial systems and still require engineering expertise to construct and program, which limits their accessibility.

As a result, for many routine tasks or field applications, researchers still resort to operating syringes by hand in small increments to avoid exceeding the container’s capacity. To bridge this gap, we have developed the *Push2Pull syringe holder*: a simple 3D-printable device that mechanically synchronizes the motion of two syringes in a push-pull configuration without the need for electronics or programming. Additionally, this device can also be used in closed systems, such as microchannels, phantom models, organ-on-a-chip, and fuel cells, to prevent pressure buildup and enable fluidic multiplexing [11], [12], [13], [14], [15], [16]. This device is easy to construct and costs less than $10 in materials, expanding the accessibility of controlled fluid handling for laboratories and field environments.

## 2 Hardware description

**Fig. 1:**
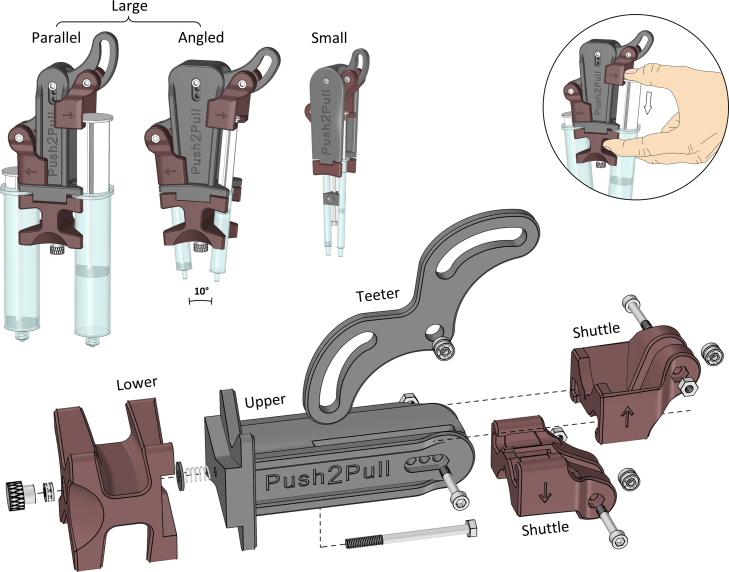
Design of the parallel, angled, and small *Push2Pull syringe holder* with 50, 5, & 1 mL syringes mounted, respectively (top). An exploded view showing how they are assembled (bottom). The syringe holder is operated with one hand: the thumb in the shuttle, and the index and middle fingers in the lower frame, as shown in the insert.

The *Push2Pull syringe holder* is designed using a computer-aided design (CAD) software (*Rhino 6*, Robert McNeel & Associates) and it exists in two different sizes, as seen in Fig. 1. The small syringe holder is made to accommodate standard disposable syringes between 1 and 10 mL (barrel diameters of 6–16 mm), while the large syringe holders are made for syringes between 3 and 60 mL (barrel diameters of 11–30 mm).

A teeter arm is used to synchronize the syringe plungers so that when one is pushed down, the other is pulled up by the same amount. By incorporating ball bearings and specific cam tracks, the teeter arm directs all the force along the syringe’s axis (as seen in Fig. S1), thereby minimizing friction and negating the need for expensive linear rails. The shape of the cam track’s lower arc is defined by the parametric equation

(1) $$\left\{\begin{array}{c} x\left(\psi \right)=\frac{c}{sin\left(\psi \right)}\cdot sin\left(cot\left(\psi \right)+\psi \right)\;\\ y\left(\psi \right)=\frac{c}{sin\left(\psi \right)}\cdot cos\left(cot\left(\psi \right)+\psi \right)\; \end{array}\right),$$

where ψ∈(π/2,π) is the parameter and c is the distance between the teeter arm’s center of rotation and the syringe’s vertical axis. This equation is also verified by comparing to a numerically envelope-of-lines simulation.

There are two versions of the large syringe holder: one that holds the syringes in parallel and one where they are angled together (10°) to reach into test tubes, and each frame has its own teeter arm. This arm can also be mounted at different heights to adjust the plunger stroke to 50, 38, or 26 mm. Additionally, there is also an extended version of the angled holder that increases the plunger stroke by 20 mm. For the small syringe holder, the frame instead includes a bottom slider for micro-adjusting the angle of the syringes (1°–5°), and it has a fixed plunger stroke of 30 mm.

This syringe holder can help researchers and lab workers in microbiology, biophysics, and biomedical engineering with:

• Medium exchange, washing, and staining at faster rates and with less risk if overflow.
• Fluidic multiplexing in microfluidics and phantom models without pressure build up.
• Operating organ-on-a-chip and fuel cells in the field.

## 3 Design files summary

All the parts files are available in the source file repository and [17], both as editable STEP-files and print-ready STL-files, as well as a version with larger manufacturing tolerances. The design files are summarized in Table 1 and a selection guide for which 3D-printed parts are required to make each syringe holder is shown below (two shuttles are needed for each). The *Push2Pull syringe holder* (Patent pending) is licensed under CC BY-NC-SA 4.0 and anyone is free to non-commercially redistribute, remix, and use the design by giving appropriate credit, while using and providing a link to the original license.

**Table:** 

Small Holder	Large Holders		
	Parallel	Angled	Angled Extended
*Upper_Small.stl*	*Upper_Parallel.stl*	*Upper_Angled.stl*	*Upper_AngledExt.stl*

*Teeter_Small.stl*	*Teeter_Parallel.stl*	*Teeter_Angled.stl*	*Teeter_AngledExt.stl*

*Lower_Small.stl*	*Lower_Parallel.stl*	———— *Lower_Angled.stl* ————	

*Shuttle_Small.stl*	———————— *Shuttle_Large.stl* ————————		

*Slider_Small.stl*			

**Table 1:** Summary of design files.

Design filename	File type	Open source license	Location of the file
Upper_Small.stl	CAD file (3D model)	CC BY-NC-SA 4.0	doi.org/10.5281/zenodo.17711432
Upper_Parallel.stl	CAD file (3D model)	CC BY-NC-SA 4.0	doi.org/10.5281/zenodo.17711432
Upper_Angled.stl	CAD file (3D model)	CC BY-NC-SA 4.0	doi.org/10.5281/zenodo.17711432
Upper_AngledExt.stl	CAD file (3D model)	CC BY-NC-SA 4.0	doi.org/10.5281/zenodo.17711432
Teeter_Small.stl	CAD file (3D model)	CC BY-NC-SA 4.0	doi.org/10.5281/zenodo.17711432
Teeter_Parallel.stl	CAD file (3D model)	CC BY-NC-SA 4.0	doi.org/10.5281/zenodo.17711432
Teeter_Angled.stl	CAD file (3D model)	CC BY-NC-SA 4.0	doi.org/10.5281/zenodo.17711432
Teeter_AngledExt.stl	CAD file (3D model)	CC BY-NC-SA 4.0	doi.org/10.5281/zenodo.17711432
Lower_Small.stl	CAD file (3D model)	CC BY-NC-SA 4.0	doi.org/10.5281/zenodo.17711432
Lower_Parallel.stl	CAD file (3D model)	CC BY-NC-SA 4.0	doi.org/10.5281/zenodo.17711432
Lower_Angled.stl	CAD file (3D model)	CC BY-NC-SA 4.0	doi.org/10.5281/zenodo.17711432
Shuttle_Small.stl	CAD file (3D model)	CC BY-NC-SA 4.0	doi.org/10.5281/zenodo.17711432
Shuttle_Large.stl	CAD file (3D model)	CC BY-NC-SA 4.0	doi.org/10.5281/zenodo.17711432
Slider_Small.stl	CAD file (3D model)	CC BY-NC-SA 4.0	doi.org/10.5281/zenodo.17711432

## 4 Bill of materials

The bill of materials has been attached to the supplementary materials, see table S1.

## 5 Build instructions

The plastic parts are designed to be 3D printed from a rigid thermoplastic polymer (e.g., ABS, PLA, PET-G, ASA, etc.) using fused deposition modeling (FDM). However, they can also be made using selective laser sintering (SLS), or even from a thermoset photopolymer using stereolithography (SLA). When using an FDM printer, the parts are strongest when printed in their original orientation, with support structures added for overhangs (<60°). We used a PLA filament (*Ultrafuse PLA*, BASF) with a *Bambu Lab X1 Carbon*, and sliced the parts in *Bambu Studio* (V1.10.0) with 0.4 mm nozzle diameter, 0.08/0.2 mm layer heights, 30% infill density, and 2 mm wall thickness. The small syringe holder used 40 g of material, while the larger holders used up to 180 g. For smooth operation of the syringe holder, we placed the Z-seam away from the bottom part of the teeter tracks and used different layer heights for the frame and shuttles.

The assembly process only requires a 2.5/3mm Allen key and is done within 10 min, see design files summary for 3D-printed parts and bill of materials for off-the-shelf parts. For the large syringe holders, start by inserting the long hex bolt through the slit in the upper frame before attaching the spring, washer, lower frame, thrust bearing, and thumb knob, as shown in Fig. 1 and S2. For the small syringe holder, the upper frame only needs a hex nut. Instead, start with the long socket head screw and mount the slider, hex nut (tighten fully), washer, lower frame, and spring before securing it to the upper frame. Slide the shuttles onto the guide rails in the upper frame and mount the teeter arm with its bearings, using a socket head screw and Nyloc nut. Move each shuttle to its top position before dropping the bearings into their cam track and attaching with a screw, tighten fully, and loosen by one turn. The thrust bearing and thumb knob thread are preferably lubricated with a plastic-compatible and high-viscosity lubricant, like silicone grease.

## 6 Operation instructions

The syringe holder can be operated with one hand just like a single syringe, as shown by the insert in Fig. 1. To start, mount the syringes with their plungers extended so that the plunger flange is situated within each shuttle (indicated with arrows). At the same time, insert the syringe barrel flange between the upper and lower halves of the frame. Once both syringes are mounted, they can be secured using the clamping knob at the bottom of the frame (an Allen key is required for the small holder). To start adding/extracting liquid, place your thumb inside the shuttle and press down on the plunger flange, while holding the lower frame by its finger rest (seen in red). The syringes can either be filled before mounting in the holder, or an exchange station can be set up using two open containers and alternating the syringe you press upon.

**Fig. 2:**
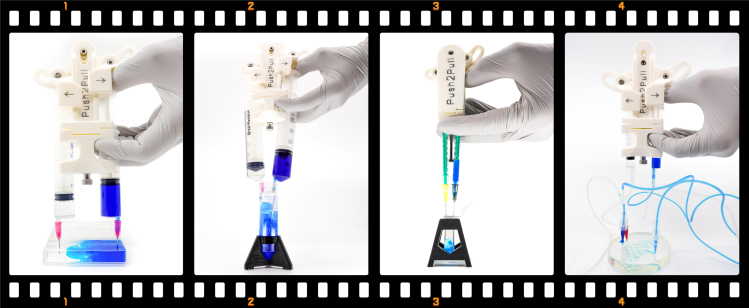
Examples of applications where the *Push2Pull syringe holder* can facilitate fluid exchanges. The first frame shows the parallel syringe holder with 20 mL syringes used to exchange liquid in a 24 mL rectangular four-well plate (*167063*, Thermo Scientific). The next frame shows the angled holder with 60 mL syringes in a 50 mL Falcon tube (*62.547.254*, SARSTEDT AG & Co. KG), followed by the small holder with 1 mL syringes in a 2 mL Eppendorf tube (*72.695.400*, SARSTEDT AG & Co. KG). The last frame shows the parallel holder with 5 mL syringes connected by hoses to a microfluidic system with more than two ports, where the passive inlet (red) is used only for observation.

## 7 Validation and characterization

The syringe holder can be used on both open and closed systems by attaching needles or tubing to the syringes. When using it with needles, the height and spacing of the needles can be adjusted to suit different applications by selecting different length needles and syringes with centered or offset Luer connectors and rotating these. As pictured in Fig. 2, the syringe holder can perform fluid exchange in a variety of different containers, as well as in microfluidic devices.

### 7.1 Exchange efficiency

**Fig. 3:**
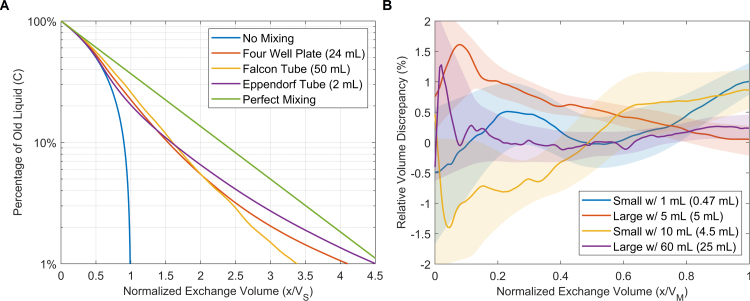
Panel A shows simulated exchange efficiency for the three open containers in Fig. 2, displayed as a percentage of old liquid remaining in the sample container (averaged over the total volume). An exchange rate of 4 $V_{S}/$min is shown here and the exchange volume is normalized to the size of each sample container $V_{S}$, i.e., $x/V_{S}=1$ when all fluid is exchanged for the theoretically best case of no mixing, while the worst case is given for perfect mixing. Panel B shows measured exchange accuracy for both the small and large (parallel or angled) syringe holders, using the min and max syringe sizes each holder can fit. The volume discrepancy is calculated relative to the volume exchanged (normalized to the max usable syringe volume $V_{M}$ shown in parentheses) and plotted as the mean of six replicates (line), along with a 95% confidence interval (shaded region). The plunger was operated slowly to avoid additional flex-induced discrepancies.

When using the simultaneous addition and extraction technique, mixing between the old and new liquids will tend to reduce the exchange efficiency. If we assume a perfect mixing during the fluid exchange, the concentration C of the old liquid in a sample of volume $V_{S}$ will decrease exponentially according to

(2) $$C\left(x\right)=C_{0}\cdot e^{-\frac{x}{V_{S}}}$$,

where $C_{0}=1$ is the initial concentration and x is the amount of fluid exchanged, i.e., the volume in the syringe. As seen in Fig. 3A (green line), using an exchange volume the same size as the sample ($x=V_{S}$) will leave 37% of the old liquid in the sample. While increasing the exchange volume fourfold ($x=4\cdot V_{S}$) will bring the final concentration just below 2%, corresponding to an exchange efficiency of 98% (defined as 1−C). However, in reality we do not have a perfect mixing, which means that the exchange rate and placement of the in- and outlet can affect the exchange efficiency a lot, e.g., operating the syringe at the right speed and choosing a syringe holder setup that reach to opposite sides of the container can help extracting the old liquid without causing excessive mixing, as evident from Fig. 2. This could thus result in a better exchange efficiency than in the perfect-mixing scenario, and hopefully approach the efficiency of the no-mixing scenario, i.e., exchanging all the old liquid at $x/V_{S}=1$ (blue line). Additionally, if the goal is to remove a diluted solute from the sample fluid, an initial concentration $C_{0}<1$ in Eq. (2) can be utilized.

To better estimate the exchange efficiency in the example applications above, we used a 3D time-dependent finite element simulation (*COMSOL Multiphysics 6.0*, COMSOL AB) to model the mixing of new and old water (at 25${}^{\circ }$C) in each sample container (filled to capacity), as detailed in the supplementary materials. The simulations were done with the *Laminar Flow* and *Transport of Concentrated Species* modules, while settling on an exchange rate of 4 $V_{S}/min$ as optimal (others seen in Fig. S3-S4). The results in Fig. 3A show similar behavior across all containers: the concentration initially follows that of no mixing, then approaches perfect mixing as most of the old liquid is exchanged. At an exchange volume of $x/V_{S}=1$ the efficiencies are all above 75%, while at $x/V_{S}=2$ the efficiencies have increased to around 94%. An efficiency of 99% is finally reached at exchange volumes of 3.3, 4.1, and 4.5 (in descending order of sample size).

### 7.2 Exchange accuracy

To test the accuracy of the syringe holder in a real-world scenario, we mounted syringes filled with water and connected them to an open container using hoses, as seen in Fig. S5. We recorded the container weight, i.e., the addition/extraction discrepancy, using a 0.1 mg analytical balance (*AE240* & *Option 011*, Mettler-Toledo GmbH) while slowly operating the plunger by hand. We tested four different syringe sizes, corresponding to the smallest and largest syringes for each syringe holder. In Fig. 3B, the discrepancy was calculated relative to the exchange volume and plotted as the mean of six replicates, along with a 95% confidence interval. The relative discrepancy falls within ±2% v/v for the whole range, with the highest relative error at low exchange volumes, i.e., short strokes of the syringe plunger. The discrepancy increased momentarily with the force applied to the plunger, which we attribute to a flex in the rubber seal of the syringe plunger. For some syringes, the maximum usable volume $V_{M}$ (in parentheses) is limited by the holder to less than the nominal volume of the syringe.

### 7.3 Conclusion

The *Push2Pull syringe holder* provides a robust, purely mechanical solution for controlled fluid exchange in open containers, such as culture plates and test tubes, while also enabling fluidic multiplexing in closed systems. Validation through simulation and experimental testing demonstrates the device’s accuracy and efficacy, achieving a 94% exchange efficiency at an exchange volume only twice the sample size, and a volumetric accuracy within ±2% across the full travel range. By utilizing the high precision and low barrier to entry provided by current 3D printers, this design is perfectly suited for on-demand manufacturing in the lab at a material cost under $10, promoting widespread accessibility. Ultimately, this device serves as a reliable and cost-effective alternative to electronic syringe pumps for simpler tasks in both laboratory and field environments.

## CRediT authorship contribution statement

**Daniel P.G. Nilsson:** Writing – review & editing, Writing – original draft, Visualization, Validation, Resources, Methodology, Conceptualization. **Magnus Andersson:** Writing – review & editing, Supervision, Funding acquisition.

## Funding

This project is financially supported by the Swedish Foundation for Strategic Research (RMX18-0152) and the Swedish Research Council (2023-04085).

## Declaration of competing interest

The authors declare the following financial interests/personal relationships which may be considered as potential competing interests: Daniel P.G. Nilsson has a pending patent application (SE 2530610-1) for the design principles of the Push2Pull syringe holder, but no other financial investments. Magnus Andersson has no conflicts of interest.

## Appendix A Supplementary data

MMC S1 The supplementary material includes diagrams showing the principle of operation and step-by-step build instructions, additional information on the simulation and experimental setups, as well as the bill of materials for both the small and large syringe holder. MMC S1
