# Supplementary material for: 3D-printed syringe holder with synchronized push-pull action
Source: HardwareX. 2026 Mar 4;25:e00756. doi: 10.1016/j.ohx.2026.e00756 (PMC12992083; doi:10.1016/j.ohx.2026.e00756)
Supplement: MMC S1 — The supplementary material includes diagrams showing the principle of operation and step-by-step build instructions, additional information on the simulation and experimental setups, as well as the bill of materials for both the small and large syringe holder. [file mmc1.pdf]

Supplementary Materials

3D-printed Syringe Holder with Synchronized Push-Pull Action

Daniel P.G. Nilsson[1], Magnus Andersson[1,2]*
[1]Department of Physics, Umeå University, Umeå 901 87, Sweden.
[2]Umeå Centre for Microbial Research (UCMR), Umeå University, Umeå 901 87, Sweden.
*Corresponding author: magnus.andersson@umu.se

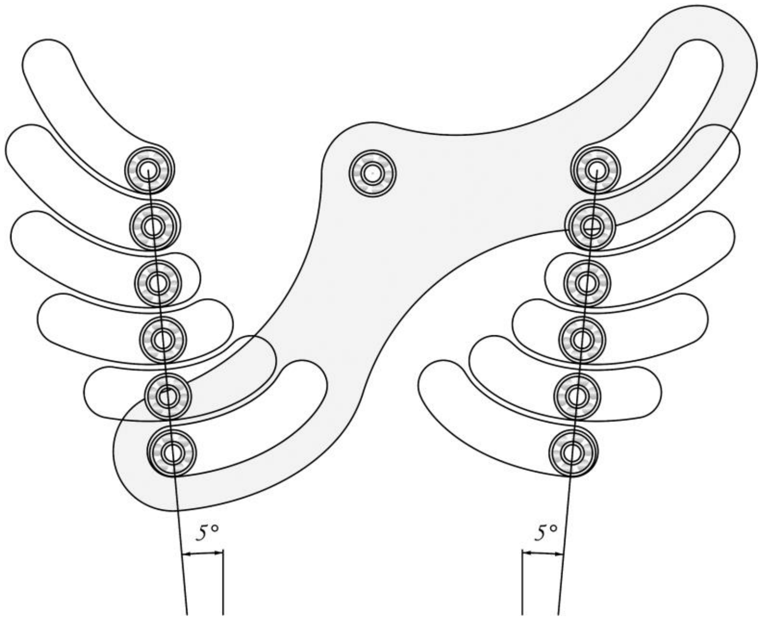

Fig. S1 The principle of operation is a teeter arm that includes a centered roller bearing and two outer cam tracks, designed so that the point of contact between the lower track and a roller bearing attached to the upper part of the shuttle is in the stable minimum position of the track and directs the pulling force along the direction of travel of the syringe plunger throughout the entire movement.

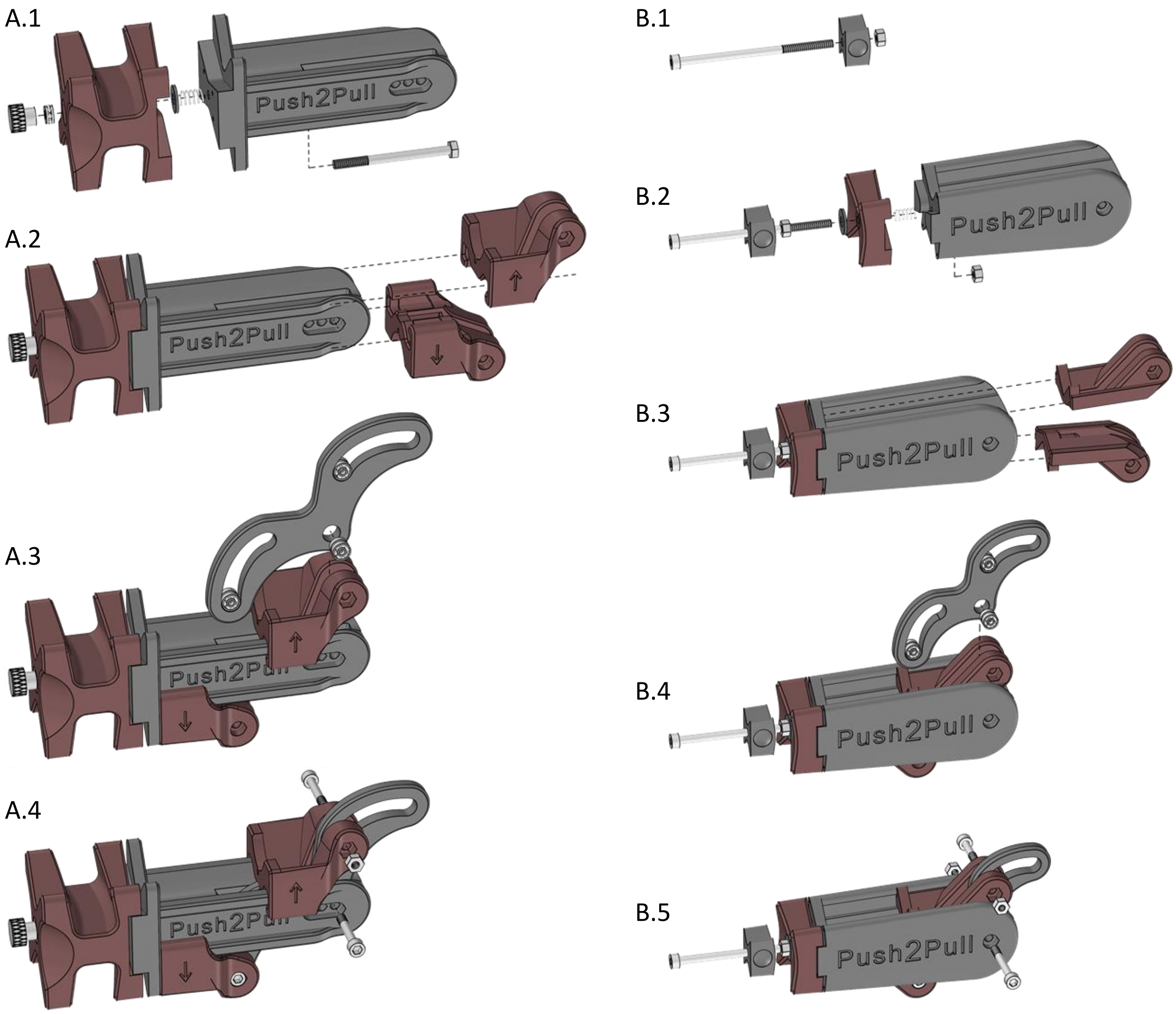

Fig. S2 Step-by-step build instruction for the large (A) and small (B) *Push2Pull* syringe holders. The dashed lines show the axes of insertion. Note that each shuttle must be moved to its top position before dropping the bearings into their cam track and attaching with a screw, then tighten fully and loosen by one turn.

CFD Simulations of Fluid Exchange

3D time-dependent computational fluid dynamics (CFD) simulations were performed in *COMSOL Multiphysics 6.0*, using the *Laminar Flow* and *Transport of Concentrated Species* modules. The simulations considered three full sample containers, without taking the free surface into account; a 24 mL rectangular four-well plate (*167063*, Thermo Scientific), a 50 mL Falcon tube (*62.547.254*, SARSTEDT AG & Co. KG), and a 2 mL Eppendorf tube (*72.695.400*, SARSTEDT AG & Co. KG), as seen in Fig. S3. The radii of the in- and outlets were fixed at 1 mm (corresponding to 12G/13G needles), and the inflow speed was set to achieve the desired exchange rate for each container. Exchange rates of 1, 2, and 4 V_S/min were tested, and the results show that higher rates promote faster mixing, whereas lower rates allow more time for self-diffusion. However, the higher flow velocities also help reduce the size of boundary layers and dead zones, thanks to the presence of large-scale vortices, and these effects appeared to be of great importance for liquid exchange in the larger containers. As such, we see no benefit of using an exchange rate much lower than 4 V_S/min, as seen in Fig. S4. At this exchange rate, the maximum Reynolds number was recorded at 10^3 by the inlet of the Falcon tube, just below the transition from laminar to turbulent flow for free jets. Further increasing the flow rate could instead introduce additional turbulent diffusion, thereby increasing mixing and lowering exchange efficiency. However, this could not be examined using the *Laminar Flow* module.

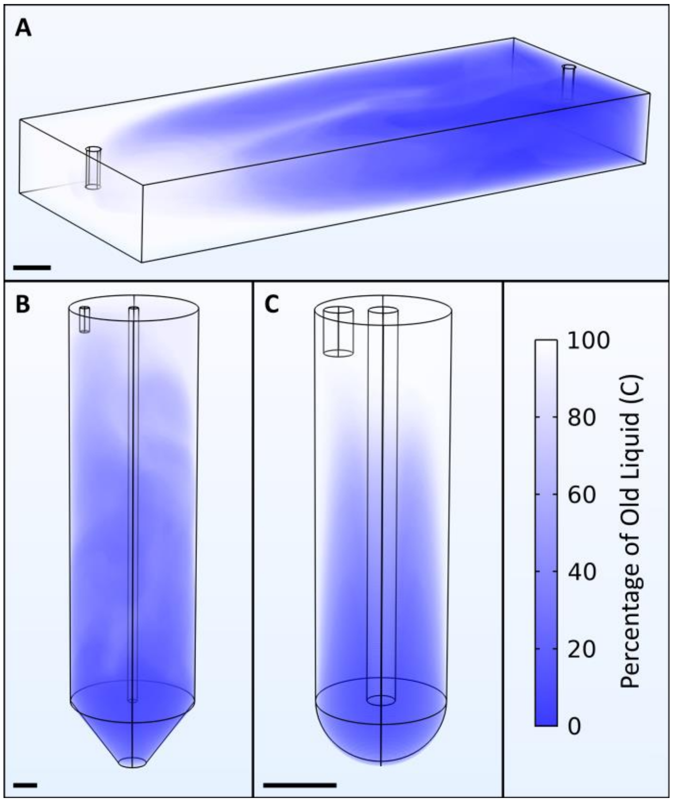

Fig. S3 Results from liquid exchange simulations of three sample containers (equivalent to Fig. 2). Panel A shows a 24 mL rectangular four-well plate, panel B a 50 mL Falcon tube, and panel C a 2 mL Eppendorf tube (with scalebars at 5 mm). The snapshots show the distribution of old liquid remaining in the sample after half of the sample volume had been exchanged ($x/V_S = 0.5$), while using a constant exchange rate of 4 V_S/min (flowing from right to left).

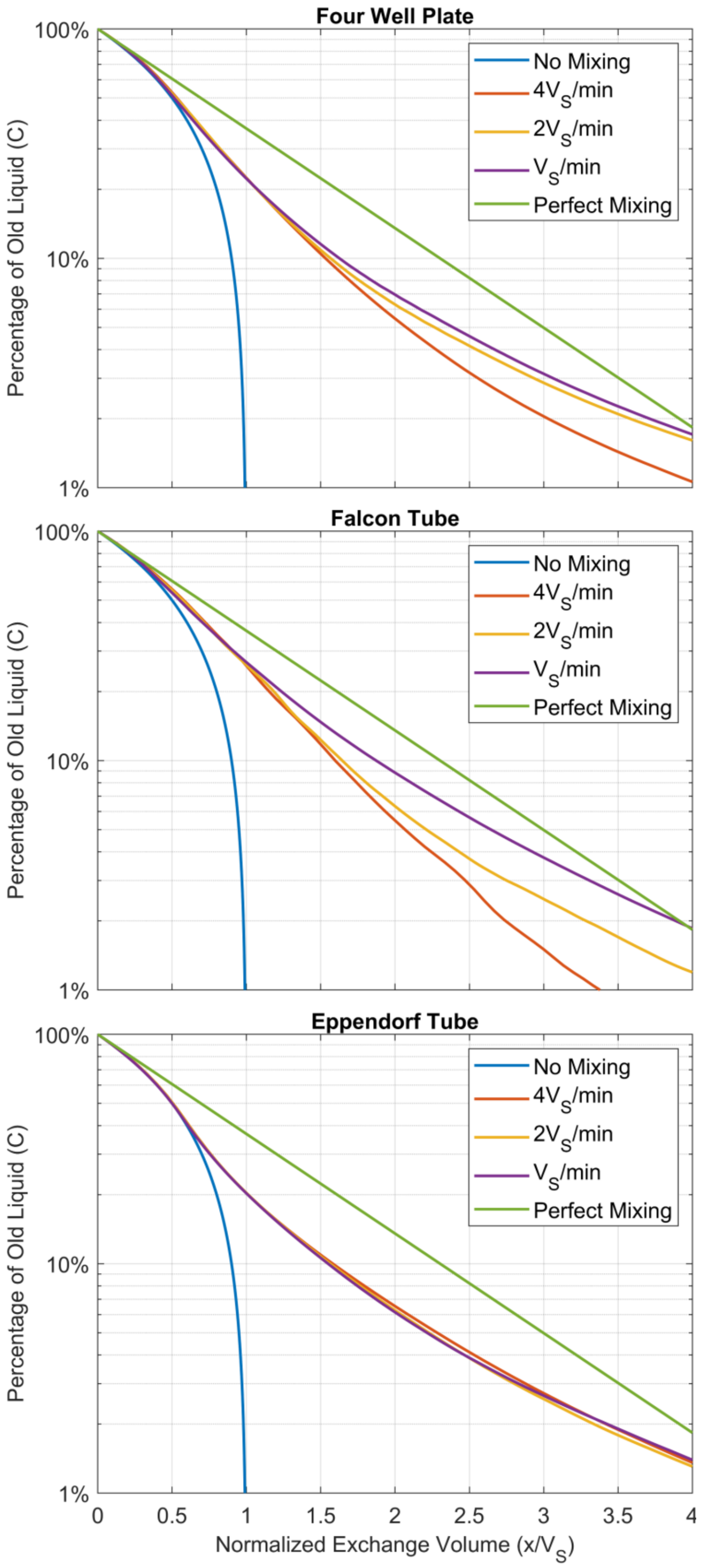

Fig. S4 Average concentration in each container and at three different exchange rates, as well as the theoretical cases. The exchange volume *x* is normalized to the size of each container V_S.

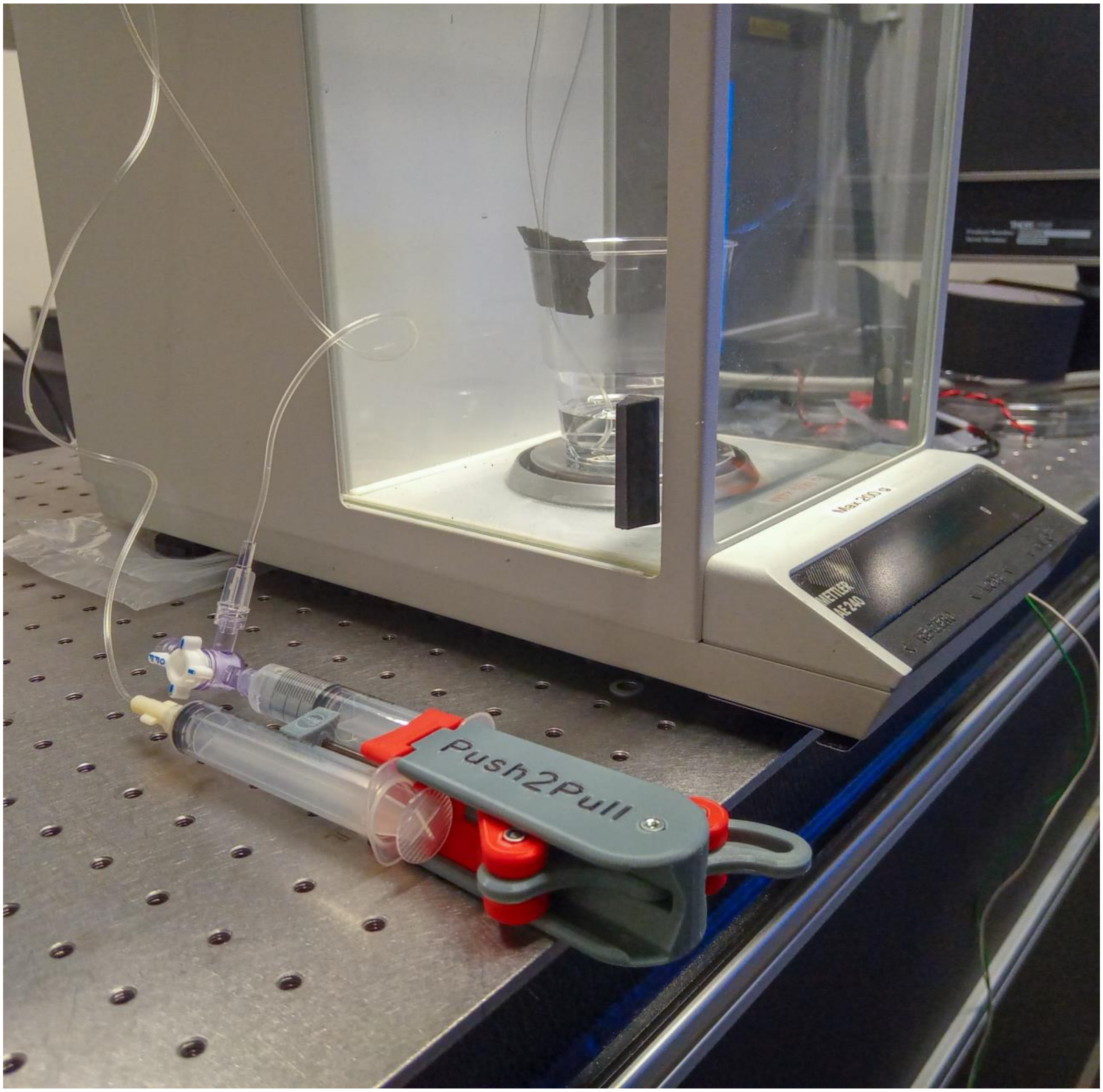

Fig. S5 Testing the exchange accuracy of each *Push2Pull* syringe holder by weighing the fluid discrepancy between the added and extracted amount, using a 0.1 mg analytical balance and a data recorder (*AE240 & Option 011*, Mettler-Toledo GmbH). Any air was bled from the system before operating the plunger slowly by hand.

Supplementary Materials

Table. S1 Bill of materials for the small (S) and large (L) syringes holders, with a final material cost of $4 and $10, respective.

Designator	Component	No.	Unit Cost ($)	Total ($)	Source	Material
S Bearing	MR63 Sealed Ball Bearing	6	0.3	1.8	aliexpress.com/item/1005002313328984.html	Metal
S Screw	M3x60 mm Socket Screw	1	0.3	0.3	aliexpress.com/item/1005008952546224.html	Metal
S Screw	M3x20 mm Socket Screw	1	0.05	0.05	aliexpress.com/item/1005007593838226.html	Metal
S Screw	M3x16 mm Socket Screw	2	0.05	0.1	aliexpress.com/item/1005007593838226.html	Metal
S Nut	M3 Nyloc Nut	3	0.05	0.15	aliexpress.com/item/4001007279182.html	Metal
S Nut	M3 Hex Nut	2	0.05	0.1	aliexpress.com/item/32977174437.html	Metal
S Washer	M3 Flat Washer	1	0.05	0.05	aliexpress.com/item/1005007189912344.html	Metal
S Spring	0.3x4x10 mm Com. Spring	1	0.1	0.1	aliexpress.com/item/1005002654047705.html	Metal
L Bearing	MR84 Sealed Ball Bearing	6	0.3	1.8	aliexpress.com/item/1005002313328984.html	Metal
L Bearing	BA4 Thrust Ball Bearing	1	0.5	0.5	aliexpress.com/item/4000219406096.html	Metal
L Screw	M4x60 mm Hex Head Bolt	1	0.3	0.3	aliexpress.com/item/32968601031.html	Metal
L Screw	M4x25 mm Socket Screw	3	0.15	0.45	aliexpress.com/item/1005007593838226.html	Metal
L Knob	M4x10x10 mm Blind Knob	1	0.7	0.7	aliexpress.com/item/1005003321136986.html	Metal
L Nut	M4 Nyloc Nut	3	0.05	0.15	aliexpress.com/item/4001007279182.html	Metal
L Washer	M4 Flat Washer	1	0.05	0.05	aliexpress.com/item/1005007189912344.html	Metal
L Spring	0.4x5x10 mm Com. Spring	1	0.1	0.1	aliexpress.com/item/1005001408323096.html	Metal
3D Filament	Ultrafuse PLA PRO1	40-180g	0.03/g	1.2-5.4	3dprima.com/basf-ultrafuse-pla_28687_10425	Polymer
